# Continuous Glucose Monitoring in Endurance Athletes: Interpretation and Relevance of Measurements for Improving Performance and Health

**DOI:** 10.1007/s40279-023-01910-4

**Published:** 2023-09-02

**Authors:** Mikael Flockhart, Filip J. Larsen

**Affiliations:** The Department of Physiology, Nutrition and Biomechanics, The Swedish School of Sport and Health Sciences, GIH, 114 33 Stockholm, Sweden

## Abstract

Blood glucose regulation has been studied for well over a century as it is intimately related to metabolic health. Research in glucose transport and uptake has also been substantial within the field of exercise physiology as glucose delivery to the working muscles affects exercise capacity and athletic achievements. However, although exceptions exist, less focus has been on blood glucose as a parameter to optimize training and competition outcomes in athletes with normal glucose control. During the last years, measuring glucose has gained popularity within the sports community and successful endurance athletes have been seen with skin-mounted sensors for continuous glucose monitoring (CGM). The technique offers real-time recording of glucose concentrations in the interstitium, which is assumed to be equivalent to concentrations in the blood. Although continuous measurements of a parameter that is intimately connected to metabolism and health can seem appealing, there is no current consensus on how to interpret measurements within this context. Well-defined approaches to use glucose monitoring to improve endurance athletes’ performance and health are lacking. In several studies, blood glucose regulation in endurance athletes has been shown to differ from that in healthy controls. Furthermore, endurance athletes regularly perform demanding training sessions and can be exposed to high or low energy and/or carbohydrate availability, which can affect blood glucose levels and regulation. In this current opinion, we aim to discuss blood glucose regulation in endurance athletes and highlight the existing research on glucose monitoring for performance and health in this population.

## Key Points

**Table:** 

Continuous glucose monitoring (CGM) has emerged as an easily assessable physiological metric and has come into use by endurance athletes with the objective of gaining insight into dietary needs and monitoring recovery and training load.
Endurance athletes represent a unique group and have repeatedly been shown to exhibit glucose profiles and responses to exercise and diet that challenge our understanding of glucose regulation.
Further research is therefore warranted to determine the value of CGM when providing assistance to endurance athletes in their pursuit to optimize training and competition outcomes, and manage a healthy approach to training.

## Glucose: A Circulating Fuel

The pool size of blood glucose is about 4 g, which is less than one percent of the stored amount of carbohydrates in humans, and the concentration is mostly kept within a range of 4–8 mM in subjects with normal glucose control [1]. Blood glucose homeostasis is highly prioritized, and the concentration is regulated through a balanced release and uptake by organs and tissues, mainly stimulated by the hormones insulin and glucagon, as well as muscle contraction. The skeletal muscles are the main site for glucose disposal during fed conditions and exercise. During unfed conditions, glucose homeostasis is regulated by the liver, which stores glycogen and can, upon stimulation, release glucose through glycogenolysis, or other substrates such as lactate, fatty acids, ketone bodies, and amino acids through gluconeogenesis. In later years, the importance of glucose as the main circulating metabolic fuel has been challenged as lactate has been recognized as a preferred fuel for oxidative metabolism in various tissues and organs [2–5]. Circulating lactate has been shown to be the main precursor to hepatic glucose production during rest [2], and plasma lactate availability to be rate limiting for gluconeogenesis during exercise [6]. Consequently, skeletal muscle tissue can affect blood glucose homeostasis not only by glucose import but also by lactate export.

## Endurance Athletes Spare Glucose During Exercise

Skeletal muscle glucose uptake can increase up to 50-fold during exercise compared with rest. This is mediated by increases in blood flow and capillary recruitment, blood glucose concentration, and muscle contraction [7, 8]. During contraction, increased metabolic activity and signaling events initiated by several pathways stimulate the glucose transporter 4-protein (GLUT4) to translocate to the cell membrane, which increases the permeability to glucose [9]. GLUT4 is considered the main controller of glucose uptake in skeletal muscle, and is most abundant in slow twitch oxidative fibers; endurance athletes have consistently been reported to have high levels of the protein [8]. To secure blood glucose homeostasis during exercise, hepatic glucose output also increases. During exercise at low intensity, this is regulated primarily through a decrease in insulin secretion, which alters the insulin/glucagon ratio and affects the liver’s sensitivity to glucagon. During high-intensity exercise, hepatic glucose output is stimulated by an increase in circulating catecholamines [7, 10, 11]. This response appears to be more substantial in endurance trained than in untrained subjects [12]. The rate of hepatic glucose output during high-intensity exercise has been shown to exceed the rate of glucose uptake, thereby increasing the concentration in the blood [11]. Intermittent exercise can further increase blood glucose levels as muscle glucose uptake is sharply lowered at the cessation of work [10], whereas hepatic glucose production appears to be adjusted with a delay [11].

Endurance athletes are known to utilize less glucose for oxidation than untrained subjects when exercising at a fixed work rate. During low-to-moderate exercise intensities, these differences are partly related to the higher oxidative capacity in endurance athletes that allows for increased use of fatty acids as substrate for ATP production, but also to reduced glycogenolysis [13, 14]. Interestingly, muscle glucose uptake in endurance athletes during moderate to high exercise intensities has been found to be inversely correlated to muscle citrate synthase activity [15] and abundance of GLUT4 [16]. Consistent with these findings, we have found that endurance trained subjects have significantly elevated blood glucose levels during a high-intensity interval training (HIIT) session, whereas the blood glucose levels of a nonendurance trained control group were unaltered compared with before HIIT [17]. We have several observations in elite athletes of capillary blood glucose > 10 mM after incremental exercise tests to exhaustion (unpublished data), highlighting that elite athletes have a glucoregulatory response to exercise that differs from the healthy recreationally active subjects. Conclusively, hyperglycemia can be expected during high-intensity exercise in endurance athletes.

## Carbohydrate Supplementation Protects Blood Glucose Homeostasis During Exercise

Although endurance athletes are adapted to maintain sufficient blood glucose levels during long-duration exercise, strenuous exercise sessions and competitions can cause hypoglycemia. Carbohydrate availability was recognized early to be important for endurance performance. In 1920, Krogh and Lindhard demonstrated that subjects exercising on a carbohydrate-rich diet experienced less fatigue than on a high-fat diet [18]. A few years later, Levine and colleagues measured blood glucose in runners after completing the Boston marathon. Many of the runners were found to have hypoglycemia, and the severity of hypoglycemia to be associated with reduced well-being and performance [19]. Therefore, during the next year’s marathon in 1925, the runners were provided with carbohydrates during the race to avoid incidences of hypoglycemia. This strategy turned out to be successful as normal blood glucose levels were observed at the finish along with increased well-being and performance [20]. On this basis, it is often assumed that low blood glucose levels are associated with fatigue, although some studies have found fatigue and low blood glucose levels to be dissociated [21, 22]. Furthermore, continuous glucose infusion has been shown to both protect performance and stabilize blood glucose levels at the end of long-term endurance exercise [23]. It has also been demonstrated to not affect performance during shorter durations of exercise [24].

The ergogenic properties of carbohydrates are also believed to be through central stimulation [25]. In several studies, mouth rinsing with carbohydrates during exercise testing has improved performance without a caloric contribution [26], and thus without affecting blood glucose levels [27]. Still, a carbohydrate-rich diet, as well as sufficient carbohydrate intake during exercise is recommended for optimizing endurance performance [28] and preserving euglycemia [29]. In this context, it is worth noting that the ability to regulate blood glucose levels under demanding conditions can constitute an important adaptation to endurance training [13]. One of the current trends in endurance sports is to optimize carbohydrate intake for maximal exogenous carbohydrate oxidation and thereby improving performance [30]. The underlying arguments are, however, related to carbohydrate availability and glycogen sparing, rather than maintaining blood glucose homeostasis. While the short-term effects of high, or even excessive, carbohydrate availability during exercise have been investigated, the long-term adaptations are unknown. Therefore, carbohydrate supplementation during exercise is recommended to be adjusted to exercise duration and intensity in a balanced manner [30].

## Hypo- and Hyperglycemia in Endurance Athletes

Recent studies in free-living endurance athletes have shown that frequent episodes of hypo- and hyperglycemia are common [31, 32]. These findings stand in contrast to the general notion that exercise training has exclusively positive effects on glucose regulation [33]. Furthermore, endurance athletes are compared with untrained subjects known to have a high capacity for glucose disposal in relation to insulin secretion [34] and show higher glucose disposal rates during lipid infusion [35]. However, athletes perform demanding exercise sessions, which have the potential to reduce glucose tolerance acutely after exercise [12, 36, 37] as well as the day after exercise [17, 38]. A plausible reason that endurance athletes sometimes are found to have reduced glucose tolerance after demanding exercise sessions is the increased lipid oxidation and transport associated with prolonged exercise and energy deficits [17, 38, 39]. As demonstrated by Ivy and colleagues, a high intake of carbohydrates after exercise has the potential to reduce this effect by suppressing lipid metabolism [37, 40]. Indeed, it is well recognized that endurance athletes have increased lipid storage in the musculature, which is often described as a paradox as this is commonly observed in insulin-resistant subjects [41–44]. That having an increased capacity for oxidation and availability of lipids as metabolic substrates may also have “paradoxical” consequences on glucose regulation is, however, not often recognized or discussed [17, 36, 38, 45]. Interestingly, it was recently shown that subjects with a higher expression of slow-twitch type I muscle fibers had better insulin sensitivity than subjects with a higher expression of fast-twitch type II fibers [46]. This suggests that not only chronic endurance training, but also fiber-type distribution, can affect glucoregulation in this population.

**Fig. 1:**
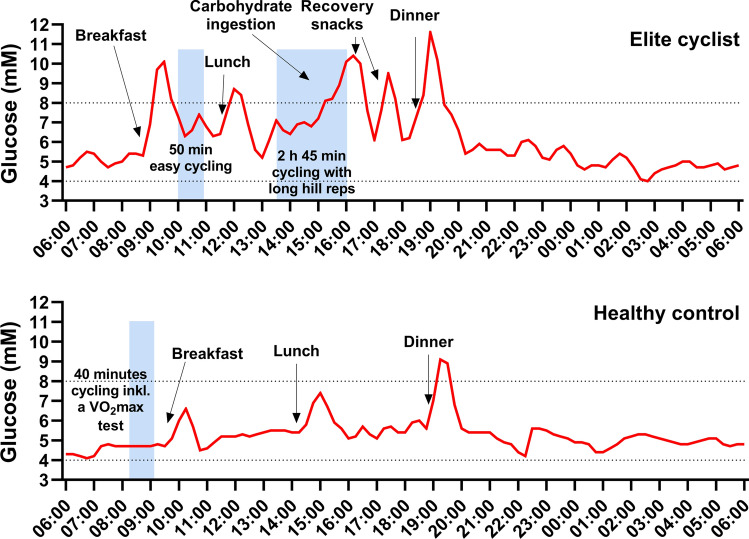
A representative 24 h segment from continuous glucose monitoring (CGM) in a male elite cyclist (*V*O_2_max 80 ml kg^−1^ min^−1^) exposed to high training load, and a healthy male control (*V*O_2_max 49 ml kg^−1^ min^−1^), showing the interstitial glucose responses to diet and exercise. CGM was assessed in the elite cyclist during a training camp, and a high carbohydrate-high caloric diet was practiced. The cyclist exhibited normal daily interstitial glucose during low training load, had normal glucose tolerance at several oral glucose tolerance tests on other occasions, as well as having a HbA1C of 5.3%. During the training camp, the cyclist performed several personal best efforts (power output for various durations) and was in excellent shape. The healthy control performed strength training on a regular basis and had an active lifestyle. CGM took place while participating in a study. The subject performed a submaximal and maximal test on a cycle ergometer in the morning and thereafter consumed a mixed diet. Note the absence of increased interstitial glucose during maximal exercise (also verified in capillary blood). The *x* axis shows the time of day, and the blue area marks the time of exercise. The normal glucose range between 4 and 8 mM is indicated with dotted lines

Endurance athletes normally plan their training sessions before larger meals and the combined effects of exercise and large intake of carbohydrates post-exercise certainly have the potential to induce hyperglycemia. In subjects that do not exercise regularly, having a hyperglycemic response after a meal can be considered a sign of reduced glucose tolerance, and having chronic high glucose levels can in the long term be detrimental to health and increase the risk for cardiovascular disease [47] and blunt adaptations to exercise training [48]. There is however no evidence that having occasional hyperglycemia might have negative effects on endurance athletes’ health in the long term. Instead, aging athletes appear to be protected against developing metabolic diseases [49] and master athletes have been shown to have lower fasting blood glucose levels than the general population [50]. Also, previous research assessing 24 h blood glucose levels in endurance athletes that had a high carbohydrate intake and performed high volumes of training, found no difference in mean plasma glucose levels compared with healthy controls (7.4 and 7.3 mM, respectively) [51]. Likewise, in work by our group, mean 24 h glucose measured with continuous glucose monitoring (CGM) was not found to be different between elite endurance athletes and a group of weight, age, and gender-matched controls performing recreational training (5.5 and 5.5 mM, respectively) [31]. In a study by Thomas and colleagues that investigated daily glucose patterns in athletes, mean 24 h interstitial glucose was not reported but can be estimated to a group mean of ~ 5.9 mM [32]. Although mean glucose levels were found normal in the two latter studies, the endurance athletes were found to spend a considerable amount of time outside the normal range of 4–6 [32] or 4–8 mM, with frequent episodes of hyperglycemia in response to meals, and in some cases hypoglycemia during late nighttime [31]. To further illustrate the high-amplitude deviations in interstitial glucose that we regularly observe in high-performing endurance athletes, we put together a figure showing the exaggerated glucose responses to exercise and diet in an elite cyclist and, for comparison, a healthy control. Without other information than the CGM curve, the elite cyclist that serves as an example can appear glucose intolerant. With additional information at hand, we instead observe a transient episode with altered glucose variability during very high training load and energy intake, (Fig. 1).

## Low energy Availability can Reduce Blood Glucose Levels

The concept of relative energy deficiency in sports (REDs) has gained an increasing amount of attention [52]. Besides inducing hormonal changes that potentially could affect sports performance and the health of the athlete, a negative energy balance has direct metabolic consequences with reduced dietary carbohydrates that enter the circulation, increased fatty acid oxidation, and gluconeogenesis. In a study where daily energy availability during 5 days was reduced by 33–78% compared with an energy balanced state, sedentary female subjects showed profound reductions in nocturnal plasma glucose levels, which in turn caused reduced mean 24 h plasma glucose levels. The lowest mean glucose levels were observed during the most severe energy deficit [53]. Also, female endurance athletes with secondary functional hypothalamic amenorrhea, which can be a consequence of low energy availability, have been found to exhibit lower capillary glucose levels during fasting and submaximal exercise than eumenorrheic subjects [54]. These, and other studies previously discussed by Bowler and colleagues [55] suggest that glucose measurements can be used as a marker of reduced energy availability.

## Hypoglycemia Might Disturb Sleep

Reduced levels of fasting glucose have previously been suggested to be a marker of overreaching in elite athletes [56] and, as previously mentioned, indicate low energy/carbohydrate availability. Hypoglycemia during the night has the potential to disrupt sleep, which could affect recovery negatively. The release of epinephrine begins already at blood glucose concentrations around 3.6–3.9 mM and intensifies as blood glucose concentration lowers [57]. Blood glucose levels below 2.8 mM have been shown to result in autonomic and neuroglycopenic symptoms and trigger an awakening response through the release of epinephrine [58]. This awakening response has been shown in several studies to dramatically decrease sleep efficiency and increase wake time during the night [59]. However, it remains to be shown if the frequent nocturnal hypoglycemia seen in athletes (own data in current projects and [31]) also affect their sleep.

## Surveilling Interstitial and Blood Glucose During Exercise and Recovery

The use of CGM has expanded in recent years and the pro and cons of the method as well as the technique has been discussed with the healthy athlete in mind [55, 60, 61]. CGM has made it possible to assess glucose variability during daily life (calculations can be found in [62]) and time in the hyper- and hypoglycemic range can easily be calculated. Studies that have implemented various forms of exercise in untrained and diabetic populations have indeed found that exercise reduces daily glucose variability and improves glucose control [63]. The interpretation of CGM data in endurance athletes is however still unclear, and it has been questioned whether the objectives of controlling glucose variability to optimize performance are possible using CGM data alone [61].

Using CGM readings to decide acute carbohydrate intake for managing blood glucose homeostasis also has some substantial limitations. Although interstitial glucose has been shown to represent the concentrations in the blood, variation can occur [64]. After carbohydrate intake, interstitial glucose increases with a delay of up to 15 min compared with concentrations in the blood, while during exercise, changes in interstitial glucose seems to occur more rapidly than in blood (personal observations and [65]). A recent study using CGM also demonstrated that individual variability of postprandial glucose responses to identical meals was as large as responses to different meals in two nondiabetic cohorts [66], indicating that additional factors in combination with food intake affect the glucose response. In addition, a lower precision of CGM measurements during exercise has been reported in type I diabetes subjects [67] and in subjects with normal glucose regulation [68]. Different sensors [69], and sites for sensor placement have also been shown to affect intestinal glucose after a glucose load, with sensors placed on the leg consistently reporting lower values than sensors placed on the upper arm during rest and when blood flow was elevated by heat exposure [70]. In all, blood and compartmental glucose are not the same, and the difference between the two measures increases when interstitial glucose fluxes change due to carbohydrate intake, blood flow, and muscular work.

Only a few scientific studies have used CGM in endurance athletes. The objectives have varied, and include surveillance of recovery [71], energy status/carbohydrate availability to avoid deficits [32], glucose responses during low energy availability [55], glucose availability during endurance exercise [72], and ultra-endurance competitions [73, 74], to investigate possible effects on glucoregulation by high training loads [31] and to describe metabolic alterations in athletes after adapting to diets high in carbohydrates or fat [75]. Glucose variability and nocturnal basal glucose have also been suggested to increase as an inflammatory response to a strenuous exercise session and potentially be used as a marker of recovery [71]. Some general conclusions can be drawn from these studies. Firstly, endurance exercise affects glucose variability and the glucoregulatory response to meals, and secondly, endurance athletes are in general prone to exhibit hyperglycemia [31, 32, 75]. Consistent with this, we have observed higher mean glucose variability (% CV) in endurance athletes (21 ± 4), than in healthy controls (16 ± 3) (calculated from the data in [31]). However, the study by Prins and colleagues show that different compositions of macronutrients in the daily diet can have a high impact on 24 h interstitial glucose and glucose variability, and that these changes are closely linked to alterations in fat metabolism [75]. Likewise, a recent study with > 7000 nondiabetic subjects showed a positive correlation between carbohydrate intake and CGM measured glucose variability (mean % CV of 16 ± 4) [62]. Interpreting CGM data can therefore be challenging, as it involves separating the effects of exercise from the effects of the timing and composition of meals. Despite this, CGMs hold promise as a tool for monitoring glucose variability, energy balance, and recovery status in endurance athletes. The daily life of an endurance athlete contains all the ingredients that can increase glucose variability, including exercise, high or low carbohydrate intake, and stress responses. This underlines the importance of further research to study the complex interactions between exercise, nutrition, and glucose regulation in this cohort.

**Fig. 2:**
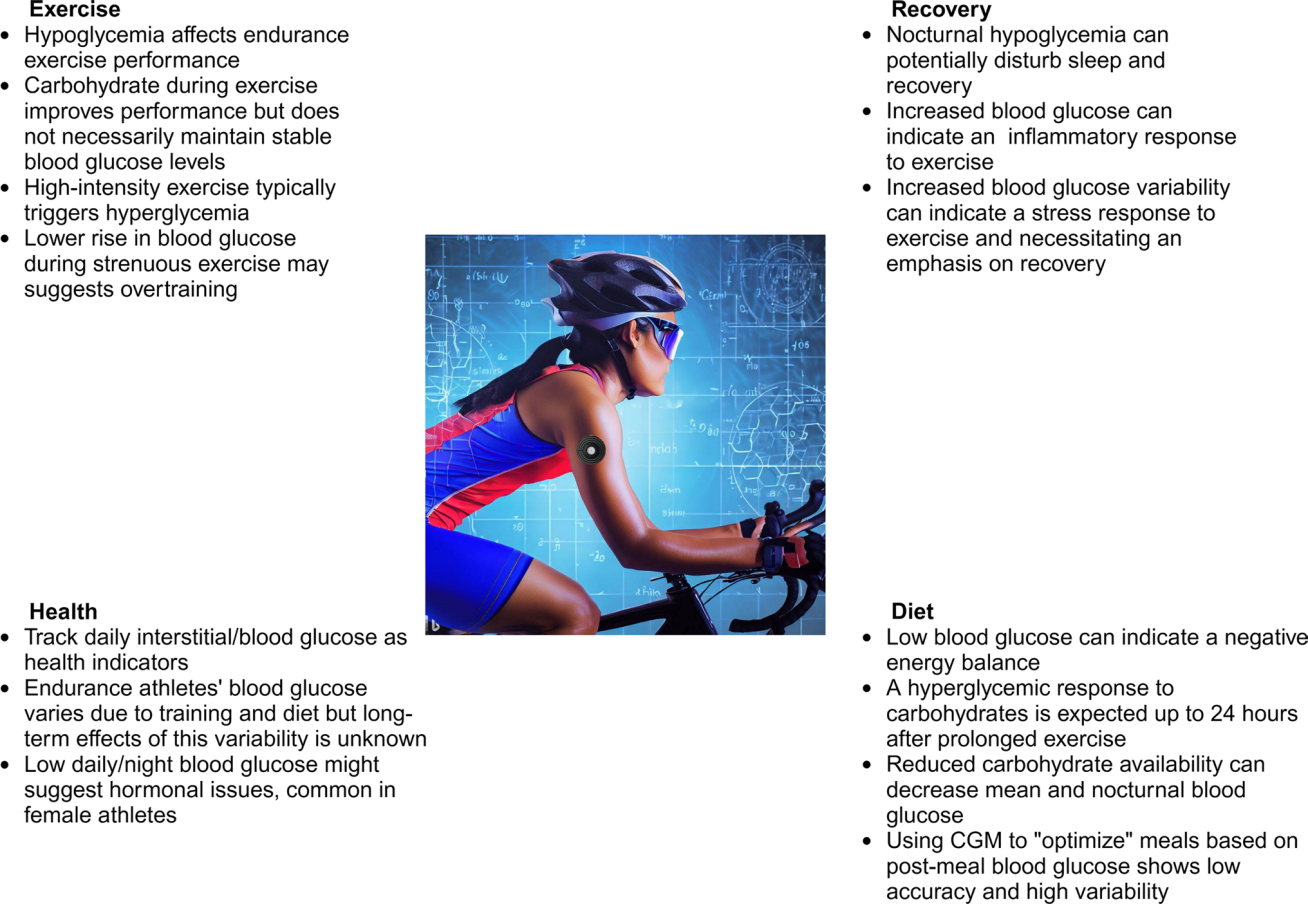
Continuous glucose monitoring (CGM) can be used by endurance athletes to track glucose levels during exercise and recovery. With knowledge of the typical glucose responses to exercise and diet in an athlete, deviations from expected glucose responses can be detected and be used to interpret levels of stress, energy availability and health. However, it should be noted that further research is needed to determine the value of CGM as a tool to better understand an athlete’s nutritional needs and to surveil the quality of recovery

While the existing studies on the relationship between blood glucose levels and athletic performance have had limitations in sample size and study design, avoiding hypoglycemia during exercise has been associated with improved performance. Suzuki and colleagues compared two runners with different training backgrounds who performed 5 h of running. Blood glucose levels were higher in the faster runner but were not affected by the timing or amount of ingested carbohydrates [72]. Ishihara and colleagues found that performance during an ultra-running event was associated with high carbohydrate intake, but normoglycemia was maintained regardless of carbohydrate intake and glucose variability was not dependent on the timing of carbohydrate ingestion [73]. In a recent cross-over study ten male athletes trained for 4 weeks while adhering to a low-carb–high-fat (LCHF), or high-carb–low-fat (HCLF) diet [75]. When training on the LCHF diet, the athletes showed profound changes with reduced mean 24 h glucose and lower nocturnal glucose with more time in the hypoglycemic range but also lower glucose variability. However, no differences were seen between groups regarding performance but during the LCHF occasion, the athletes had higher fat oxidation rates but also higher ratings of session affect (a measure of perceived training stress). Interestingly, the increase in capillary glucose after high intensity exercise was similar in the two groups, indicating that this specific response is not dependent on carbohydrate availability. This supports our argument above that accomplished endurance athletes have an improved intrinsic ability to maintain normal to elevated blood glucose during extended exercise. However, a failure to increase glucose during high-intensity exercise has been associated with maladaptations to the training load during overreaching, possibly through a reduced catecholamine response during exercise [31]. Postexercise glucose measurements have therefore the potential to be used to detect early indices of overtraining [76]. A summary of possible usage of CGM in athletes is presented in Fig. 2.

Finally, the advancements in sensor technology including other measures than glucose should be mentioned. Sensors are currently being developed for continuous dermal interstitial monitoring of not only glucose but also lactate and possibly other biomarkers relevant to the athlete [77]. A sensor that with high accuracy can track multiple physiological responses to exercise and during recovery would certainly move the field forward. One obstacle to this is that the International Cycle Union in 2021 prohibited the use of technology that captures metabolic data (which includes CGM) during competitions [78]. A common CGM sensor life is 14 days and, for example, cyclists that compete frequently can therefore not take full advantage of the technique. It is important to note that there is no evidence suggesting that the use of CGM provides an unfair advantage during competitions. Instead, such a ban may represent a missed opportunity for researchers and coaches to monitor the health of athletes.

## Conclusions and Perspectives

Blood glucose regulation is a complex process that is influenced by various factors, including dietary carbohydrates, exercise, and an individual’s training status. As such, optimizing glucose levels to improve performance and health outcomes in athletes is a growing area of practice and research. However, our knowledge of what the optimal glucose response to exercise and the following recovery period looks like is still untangled. Studies utilizing continuous glucose monitoring (CGM) have consistently shown that athletes have highly individual glucose profiles and often spend a significant amount of time with hypo- and hyperglycemia. These findings challenge our traditional assumptions about glucose control and suggest that interstitial and blood glucose levels may be an overlooked parameter in optimizing athletic performance. With the help of future researchers, athletes, and governing bodies in sports working together, we can uncover new insights into how glucose regulation affects performance, recovery, and overall health. These insights may help athletes tailor their training and nutrition plans to meet their individual needs, ultimately leading to improved performance and better health outcomes.
